# CRANIUM: a quasi-experimental study to improve metabolic screening and HIV testing in community mental health clinics compared to usual care

**DOI:** 10.1186/s12888-022-04293-4

**Published:** 2022-11-09

**Authors:** Alison R. Hwong, D. Nyasha Chagwedera, Marilyn Thomas, Grace Niu, Judy Quan, Eric Vittinghoff, Dean Schillinger, John W. Newcomer, Ana Gonzalez, Susan Essock, Christina Mangurian

**Affiliations:** 1 Department of Psychiatry and Behavioral Sciences, University of California, San Francisco, San Francisco, CA USA; 2 UCSF National Clinician Scholars Program, San Francisco Veterans Affairs Medical Center, San Francisco, CA USA; 3 UCSF School of Medicine, San Francisco, CA USA; 4 Department of Epidemiology and Biostatistics, UCSF, San Francisco, CA USA; 5 UCSF Center for Vulnerable Populations at Zuckerberg San Francisco General Hospital, San Francisco, CA USA; 6 UCSF Division of General Internal Medicine at Zuckerberg San Francisco General Hospital, San Francisco, CA USA; 7 Thriving Mind South Florida, Miami, FL USA; 8 Department of Psychiatry, Washington University School of Medicine, St. Louis, MO USA; 9 Department of Psychiatry, Columbia University, New York, NY USA; 10 UCSF Philip R. Lee Institute for Health Policy Studies, San Francisco, CA USA

**Keywords:** Integrated care, Collaborative care, Serious mental illness, Health care disparities, Diabetes

## Abstract

**Background:**

Individuals with serious mental illness often do not receive guideline-concordant metabolic screening and human immunodeficiency virus (HIV) testing, contributing to increased morbidity and premature mortality. This study evaluates the effectiveness of CRANIUM (*Cardiometabolic Risk Assessment and treatment through a Novel Integration model for Underserved populations with Mental illness)*, an intervention to increase metabolic screening and HIV testing among patients with serious mental illness in a community mental health clinic compared to usual care.

**Methods:**

The study used a quasi-experimental design, prospectively comparing a preventive care screening intervention at one community mental health clinic (*n* = 536 patients) to usual care at the remaining clinics within an urban behavioural health system (*n* = 4,847 patients). Psychiatrists at the intervention site received training in preventive health screening and had access to a primary care consultant, screening and treatment algorithms, patient registries, and a peer support specialist. Outcomes were the change in screening rates of A1c, lipid, and HIV testing post-intervention at the intervention site compared to usual care sites.

**Results:**

Rates of lipid screening and HIV testing increased significantly at the intervention site compared to usual care, with and without multivariable adjustment [Lipid: aOR 1.90, 95% CI 1.32–2.75, *P* = .001; HIV: aOR 23.42, 95% CI 5.94–92.41, *P* < .001]. While we observed a significant increase in A1c screening rates at the intervention site, this increase did not persist after multivariable adjustment (aOR 1.37, 95% CI .95–1.99, *P* = .09).

**Conclusions:**

This low-cost, reverse integrated care model targeting community psychiatrist practices had modest effects on increasing preventive care screenings, with the biggest effect seen for HIV testing rates. Additional incentives and structural supports may be needed to further promote screening practices for individuals with serious mental illness.

**Supplementary Information:**

The online version contains supplementary material available at 10.1186/s12888-022-04293-4.

## Background

Individuals with serious mental illness (SMI), such as schizophrenia and bipolar disorder, have an average life expectancy 10–20 years shorter than the general population [1]. Cardiovascular disease is the leading cause of death, likely due to higher rates of diabetes, hyperlipidaemia, and smoking in this population [1, 2]. A key contributor to the increased burden of cardiometabolic disease is limited access to, and receipt of, primary health care services [3]. Screening rates for diabetes and dyslipidaemia are consistently low for people with SMI [4–7]. This disparity exists despite national guidelines for routine monitoring of cardiometabolic risk factors for patients prescribed second-generation antipsychotics due to the cardiometabolic adverse effects of these medications [8, 9]. Though clinical practice guidelines were issued starting in 2003, a 2011 meta-analysis found that fewer than half of people taking antipsychotics received recommended routine haemoglobin A1c (A1c) and lipid screening across studies [10]. Individuals with SMI also are less likely to receive recommended human immunodeficiency virus (HIV) testing, despite higher rates of HIV risk factors, HIV prevalence, and HIV/AIDS-related morbidity [11, 12]. HIV confers additional cardiometabolic risks for this already high-risk population [13].

Publicly insured patients may face even greater barriers to accessing preventive care: in a study of California Medicaid recipients with SMI, 70% had no diabetes screening [4]. This gap in care carries financial implications, with Medicaid patients newly prescribed antipsychotics incurring $1,249 more in health care expenses due to incident cardiometabolic conditions compared to patients without cardiometabolic conditions over a three-year period. [14, 15]

Though several models to improve primary care access for people with SMI have been proposed and trialed, no single approach is yet accepted for broad-based implementation [16–19]. The largest trial to date was a behavioural health home model targeting patients with SMI and at least one cardiometabolic risk factor to improve monitoring and treatment [20]. Employing a full-time nurse care manager and onsite primary care provider in a mental health clinic, the intervention was associated with an increased receipt of preventive services, but no statistically significant changes in cardiometabolic outcomes. However, implementing a behavioural health home model requires substantial financial and structural investment from health systems, and this study did not address HIV and other infectious disease testing [21, 22]. For low-resource settings with limited capacity to hire new staff or acquire new space, a different approach may be needed.

The Cardiometabolic Risk Assessment and treatment through a Novel Integration model for Underserved populations with Mental illness (CRANIUM) study aimed to fill this gap by trialing a low-cost model of care to improve metabolic screening in a community mental health setting. The CRANIUM intervention was designed following principles of the Collaborative Care Model (CoCM), an integrated care model which offers psychiatric consultation to primary care providers in order to expand mental health care access to patients [23, 24]. Using a “reverse integrated care” approach, the CRANIUM intervention aimed to expand the scope of practice of community psychiatrists through trainings in primary care practices and easy access to electronic primary care consultation [25]. As with all integrated care approaches, the goal was to meet patients where they are already receiving care.

CRANIUM consists of four components: 1) patient-centred team-based care; 2) panel management using patient registries; 3) training and protocols on cardiometabolic screening and HIV testing for psychiatrists; and 4) training and protocols for treatment of diabetes, hypertension, and dyslipidaemia for psychiatrists [23, 26]. The objective of this study was to assess the effectiveness of the CRANIUM intervention on increasing metabolic screening and HIV testing rates compared to usual care.

## Methods

### Setting

The study used a quasi-experimental design, with the intervention implemented at one community mental health clinic and prospectively compared to usual care at the remaining community mental health clinics within a large urban public behavioural health system. Criteria for admission to the behavioural health system clinics include a SMI diagnosis; co-occurring substance use disorder, co-morbid medical issue, or cognitive impairment; frequent psychiatric acute care service utilization and/or legal system involvement; and circumstances substantiating need for intensive case management (e.g., homelessness, assaultive behaviour, adherence difficulties). The intervention clinic was chosen based on strong pre-existing relationships with the research group, an investment in improving primary care from clinic leadership, and a high proportion of patients with complex psychosocial and medical needs. The intervention clinic provides outpatient care, case management, and support services to approximately 600 publicly insured adults with SMI annually.

The usual care group continued to receive regular care in their mental health clinics and could also receive care from primary care providers in other clinics, without explicit integration of services. While providers from different specialties could access common electronic health records, patients in usual care seeking non-psychiatric medical care had to go to separate clinics and laboratories for screening without additional support.

### Study population

Participants were adults (18 and older) diagnosed with SMI and continuously enrolled in the public behavioural health system from January 2014 to December 2015. Exclusion criteria included being in jail and/or in a locked short-term care facility during the study period. This study received institutional review board (IRB) approval from the University of California, San Francisco. The IRB approved a waiver of consent for clinic patients as the recruitment procedures involved routine review of medical records, did not adversely affect the rights and welfare of participants, and posed minimal risk to subjects and their privacy.

### Intervention

Development of CRANIUM has been previously described [25]. Briefly, study researchers used the Behavioural Change Wheel model, an implementation science framework that identifies targets for behavioural intervention and has proven to be effective in patients with SMI [27]. Feedback from focus groups of psychiatrists and patients informed the design and implementation of the intervention, [25, 27] which consisted of the following four components:

### Patient-centred team-based care

CRANIUM utilized pre-existing resources in specialty mental health clinics (the psychiatrist and case manager) and added a primary care consultant at 0.1 FTE. Rather than co-locating primary care providers at a Federally Qualified Health Centre as has been attempted previously [17], the primary care provider was integrated as an electronic consultant (eConsultant), available to answer questions (e.g., medication initiation, connecting to primary care services). A peer navigator was also integrated into the team and prepared lab slips, accompanied selected patients to lab facilities, and entered results into the electronic health record (EHR).

### Panel management with patient registries

The CRANIUM registry included test results from three separate EHRs operating across the health system: mental health EHR (AVATAR), primary care EHR (Invision), and laboratory results (LabCorp Beacon). Each month, research staff extracted data from these EHRs on patients who had treatment plans due and compiled information into a single separate electronic database. The information was distributed to psychiatrists and case managers via a personalized spreadsheet. Laboratory slips were pre-completed for all identified patients and provided to the psychiatrist. The staff and research team met quarterly to conduct panel management and problem solve for those requiring screening or treatment.

### Trainings and protocols for psychiatrists on metabolic screening and HIV testing

Psychiatrists were trained to order annual screening for hypertension, A1c, total cholesterol, high-density lipoprotein (HDL), and low-density lipoprotein (LDL). Given that people with SMI are also at greater risk for HIV, but have low testing rates, [12, 28] we included annual HIV testing. Laboratory slips were pre-completed for all identified patients and provided to the psychiatrist. To promote consistent screening, psychiatrists received a monthly registry-based personalized list of patients missing labs or vital signs. For those patients who were missing labs, based upon individual need and preference, a peer navigator was available to accompany patients with missing labs to appropriate lab facilities, and an on-site nurse was available to draw labs.

### Training and protocols for treatment of diabetes, hypertension, and dyslipidaemia

To mitigate concerns previously reported by psychiatrists in prescribing non-psychotropic medications, [29] the primary care consultant provided a one-time in-person group training on guideline-recommended pharmacological management of common metabolic abnormalities to all psychiatrists. This training is now available via SMI Adviser [30]. In addition, easy-to-use, evidence-based medication algorithms were available in all treatment rooms and online. Patients with cardiovascular risk factors were highlighted to facilitate treatment discussions during panel management meetings.

The CRANIUM intervention was delivered in 2015 over twelve months at modest cost: $74 annually per patient. [31]

### Study outcomes

This report focuses on screening outcomes only. Feasibility outcomes have been published elsewhere [32]. The main outcomes of interest were individual-level screening for diabetes (A1c), fasting lipids, and HIV testing at least once in the year before and after the intervention. We did not include annual blood pressure measurement as an outcome due to the lack of reliable data (across arms, 72% missing or unmeasured outcomes in 2015). Inconsistent information about previous participant diagnoses of diabetes, hypercholesterolemia, and HIV precluded our ability to exclude people with these pre-existing conditions from the analysis. Demographics (age, sex, race/ethnicity) were also collected.

### Statistical analysis

Chi-square tests and t-tests were used to compare baseline demographics between the study arms. Because dropout information was only available from the intervention site and not from usual care sites, we conducted an intention-to-treat analysis and included all participants before and after the intervention. However, we have provided dropout information in the Supplement. Mixed effects logistic models with nested random effects for clinic and participant and including interaction term for intervention condition and time were used to estimate between-arm differences in the change from pre- to post-intervention in A1c and lipid screening and HIV testing rates, first without adjustment, then adjusting for age, sex, race, and ethnicity. A two-sided *P* value < 0.05 was considered statistically significant. The planned sample of 5,000 was estimated to provide 80% power in 2-sided tests to detect 5–6 percentage point differences between arms in the change in screening and testing rates. Estimates were obtained by approximating the covariance matrix of the coefficients estimated by the random effects logistic model used for the analysis and implemented in R (R Foundation for Statistical Computing, Vienna, Austria, 2020). Analyses were performed using Stata MP Version 15.1 (College Station, Texas).

## Results

The CRANIUM intervention included 536 participants at one clinic and 4,847 patients in usual care at six clinics. At the intervention site, 54 (10%) participants moved out of county, 28 (5%) declined screening, 22 (4%) were in a locked facility, and 7 (1%) died (see Supplemental Material). Dropout information was not available at the usual care site so all participants were include at all sites before and after the intervention. Participant characteristics at baseline are summarized in Table 1. Compared to usual care, the CRANIUM arm participants were younger, more likely to be male, and more likely to be Black.

**Table 1:** Demographics of study participants

Characteristic	CRANIUM *N* = 536		Usual Care *N* = 4,847		*P* value
	***N***	***%***	***N***	***%***	
**Age**					
Mean yrs (SD)	46.9 (11.8)		49.0 (13.6)		< .001
18–30	66	12.3%	577	11.9%	< .001
31–40	110	20.5%	824	17.0%	
41–50	149	27.8%	1116	23.0%	
51–60	145	27.0%	1393	28.7%	
61–64	40	7.5%	453	9.4%	
65 and older	26	4.9%	484	10.0%	
**Sex**					
Female	170	31.7%	2310	47.7%	< .001
**Race**					
White	202	37.7%	2159	44.5%	< .001
Asian	145	27.1%	1633	33.7%	
Black/African American	144	26.9%	611	12.6%	
American Indian/Alaska Native	9	1.7%	58	1.2%	
Native Hawaiian/Pacific Islander	5	0.9%	26	0.5%	
More than one race	15	2.8%	59	1.2%	
Missing	16	3.0%	301	6.2%	
**Ethnicity**					
Hispanic/Latino	56	10.4%	818	16.9%	< .001
Unknown	6	1.1%	232	4.8%	

Percentages may not add to 100 due to rounding

*P* values derived from chi-square tests and t-tests

**Fig. 1:**
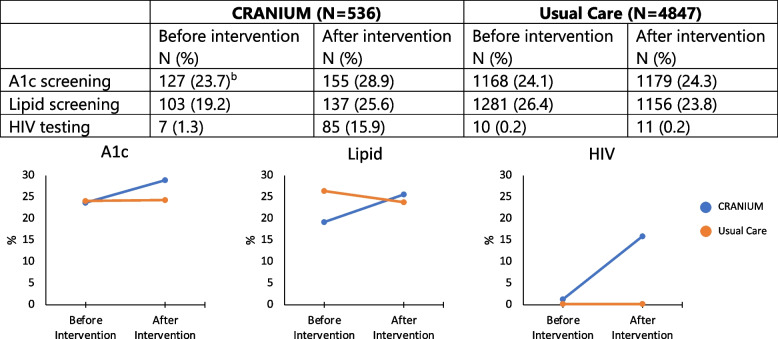
A1c screening, lipid screening, and HIV testing rates before and after intervention by intervention arm^a^ (Abbreviations: A1c: Haemoglobin A1c, HIV: Human Immunodeficiency Virus. (^a^Only the CRANIUM arm received the intervention.) (^b^No statistical significance by intervention arm for A1c screening before intervention (χ^2^ = .04, *P* = .84).) CRANIUM lipid screening before intervention is significantly lower than usual care (χ^2^ = 13.1, *P* < .001). CRANIUM HIV testing before intervention is significantly higher than usual care (χ^2^ = 18.5, *P* < .001))

Pre- and post-intervention screening rates are presented in Fig. 1. The two arms had comparable A1c screening rates at baseline (23.7% CRANIUM vs. 24.1% usual care, *P* = 0.84). However, at baseline, the CRANIUM arm had a lower rate of lipid screening and higher rate of HIV testing than usual care (Lipid: 19.2% CRANIUM vs. 26.4% usual care, *P* < 0.001; HIV: 1.3% CRANIUM vs. 0.2% usual care, *P* < 0.001).

As shown in Table 2, the CRANIUM intervention was associated with significant increases in A1c screening relative to usual care (adjusted odds ratio (aOR) 1.49, *P* = 0.03) over the same time period, but these differences did not persist after multivariable adjustment (aOR 1.37, *P* = 0.09). Rates of lipid screening and HIV testing increased significantly in the CRANIUM group compared to usual care, with and without multivariable adjustment [Lipid: aOR 1.90, *P* = 0.001; HIV: aOR 23.42, *P* < 0.001].

**Table 2:** Comparison of CRANIUM to usual care in changes in screening and testing rates^a^

CRANIUM vs. Usual Care	ORs (95% CI)^b^	*P* Value	Adjusted ORs (95% CI)	*P* Value
A1c screening	1.49 (1.04–2.13)	.03	1.37 (.95–1.99)	.09
Lipid screening	2.01 (1.41–2.87)	< .001	1.90 (1.32–2.75)	.001
HIV testing	16.16 (4.45–58.75)	< .001	23.42 (5.94–92.41)	< .001

^a^Mixed effects regression analyses with interaction between intervention and time, and nested random effects for individual and clinic, adjusted for age, sex, race, and ethnicity. *P* values given are 2-sided

^b^*OR* Odds ratio, *CI* Confidence interval

## Discussion

To our knowledge, this is the first study of a novel, low-cost reverse integrated care intervention to increase population-based metabolic screening and HIV testing in a community mental health clinic. Unlike previous trials to promote satellite primary care practices co-located in mental health settings, this approach uses pre-existing structures with minimal additional support needed and relies on the psychiatrist to take responsibility for preventative health screening. Such an approach makes it a potentially useful model to deploy in resource-limited settings in which hiring staff, acquiring more clinic space, or converting to a new electronic records system may be challenging.

In the CRANIUM arm, lipid screening and HIV testing increased compared to usual care, and a positive trend was observed for diabetes screening. Unlike previous studies that primarily focused on patients with cardiometabolic risk factors or poorly controlled diabetes, [20, 21] CRANIUM included all people with SMI seen in a community mental health setting and focused on screening as a gateway to treatment and referral rather than treatment outcomes. In addition, the CRANIUM intervention was unique from previous work in facilitating not only cardiometabolic screening—the focus of most prior studies—but also HIV testing, given the well-documented high prevalence of HIV among this vulnerable population [11]. Overall, however, the gains were modest, and may reflect that relying on busy psychiatrists to address preventive care screening may not be sufficient without other supports and incentives. Based on the findings from other population-based interventions, clinician-based training and support may need to be coupled with tying reimbursement rates to quality metrics for robust improvements in metabolic screening [33]. Additional research is needed to understand why screening rates remained low for the vast majority of individuals in the intervention clinic, and to evaluate barriers at the patient-, provider- and systems-level.

The most striking effect of CRANIUM was on rates of HIV testing, which has generally not been included in reverse integration models. Both study arms had low baseline levels of HIV testing, echoing previous findings that this is an overlooked primary care service among people with SMI [11, 28]. The greater than 12-fold increase in testing rates in the CRANIUM arm offers evidence for the feasibility of broad deployment of HIV testing in mental health clinics and is of clinical importance, though 85% of participants still lacked routine testing. Given this major gap in care identified in the usual care sites, our research team has been investigating HIV care along the continuum (including testing) in a national cohort of Medicaid recipients with schizophrenia (R01MH112420). Beyond testing, future studies are needed to develop evidence-based HIV prevention approaches tailored for this high-risk group. [34, 35]

This study has a number of limitations to consider. The implementation of the intervention in a single urban public mental health clinic limits generalizability of the findings. Despite adjusting for demographic variables, there may be unmeasured confounding that affected the variability in outcomes compared to usual care due to the quasi-experimental study design. We did not distinguish between reasons for not receiving screening; that is, patient refusal of screening was coded as non-receipt, as was non-receipt due to other reasons (e.g., never being offered screening). The intervention did not address tobacco use screening, a major contributor to premature mortality in the SMI population [36]. Although we did not collect data on individual behaviours that may elevate risk for HIV, the SMI population on average is at increased risk of sexually transmitted infections, including HIV, and thus should be routinely tested [37–39]. While the intervention and usual care arms had slightly different demographic profiles at baseline, the intervention site serves a large forensics population with high levels of co-morbid substance use, a patient group that is particularly vulnerable and in need of comprehensive primary care. Effectiveness in this setting suggests that the CRANIUM model may work in a variety of both low-resource and better resourced clinics. A multisite, cluster randomized trial would more rigorously test the effectiveness of the intervention and could not be conducted at the time due to time and funding limitations. The intervention period lasted only one year, so a longer trial is needed to assess durability of adoption and implementation. Inconsistent information about previous participant diagnoses of diabetes, hypercholesterolemia, and HIV precluded our ability to exclude them from the analysis, which could bias estimates. In addition, this study focused on process measures and did not evaluate patient health outcomes for individuals with SMI.

## Conclusion

As the morbidity and mortality gap for people living with serious mental illness persists, we must continue to develop innovative solutions to improve preventive care for this population. CRANIUM is one demonstrated model to be further refined, studied, and validated in a large cluster randomized controlled trial. Our findings from this reverse integration intervention add to the growing body of literature focused on providing primary care services in specialty mental health settings.

## Supplementary Information

**Additional file 1:** **Supplemental Material.** Flow chart of participants at intervention and usual care sites.

## Data Availability

The datasets analysed during the current study are available from the corresponding author on reasonable request.

## Abbreviations

aOR: Adjusted odds ratio
A1c: Haemoglobin A1c
CoCM: Collaborative care model
CRANIUM: Cardiometabolic risk assessment and treatment through a novel integration model for underserved populations with mental illness
EHR: Electronic health record
HDL: High density lipoprotein
HIV: Human immunodeficiency virus
IRB: Institutional review board
LDL: Low density lipoprotein
OR: Odds ratio
SMI: Serious mental illness
