# Supplementary material for: CRANIUM: a quasi-experimental study to improve metabolic screening and HIV testing in community mental health clinics compared to usual care
Source: BMC Psychiatry. 2022 Nov 9;22:687. doi: 10.1186/s12888-022-04293-4 (PMC9644536; doi:10.1186/s12888-022-04293-4)
Supplement: Supplementary file 1 — Additional file 1: Supplemental Material. Flow chart of participants at intervention and usual care sites. [file 12888_2022_4293_MOESM1_ESM.docx]

**Supplemental Material. Flow chart of participants at intervention and usual care sites.**
